# What embedded counterfactuals tell us about the semantics of attitudes

**DOI:** 10.1515/lingvan-2021-0032

**Published:** 2022-07-15

**Authors:** Nina Haslinger, Viola Schmitt

**Affiliations:** Georg-August-Universität Göttingen, Göttingen, Germany; Humboldt-Universität zu Berlin, Berlin, Germany

**Keywords:** attitude predicates, counterfactuals, epistemic modality, ordering semantics, possible-worlds semantics

## Abstract

We discuss German examples where counterfactuals restricting an epistemic modal are embedded under *glauben* ‘believe’. Such sentences raise a puzzle for the analysis of counterfactuals, modals, and belief attributions within possible-worlds semantics. Their truth conditions suggest that the modal’s domain is determined exclusively by the subject’s belief state, but evaluating the counterfactual separately at each of the subject’s doxastic alternatives does not yield the correct quantificational domain: the domain ends up being determined by the facts of each particular world, which include propositions the subject does not believe. We therefore revise the semantics of counterfactuals: counterfactuals still rely on an ordering among worlds that can be derived from a premise set (Kratzer, Angelika. 1978. *Semantik der Rede: Kontexttheorie – Modalwörter – Konditionalsätze* (Monographien Linguistik und Kommunikationswissenschaft 38). Königstein: Scriptor, 2012 [1981]a. The notional category of modality. In *Modals and conditionals* (Oxford studies in theoretical linguistics 36), 27–69. Oxford: Oxford University Press), but rather than uniquely characterizing a world, this premise set can be compatible with multiple worlds. In belief contexts, the attitude subject’s belief state as a whole determines the relevant ordering. This, in turn, motivates a revision of the semantics of *believe*: following Yalcin’s work on epistemic modals (Yalcin, Seth. 2007. Epistemic modals. *Mind* 116. 983–1026), we submit that evaluation indices are complex, consisting of a world and an ordering among worlds. Counterfactuals are sensitive to the ordering component of an index. Attitude verbs shift both components, relativizing the ordering to the attitude subject.

## 1 Introduction

This squib discusses counterfactual conditionals constraining existential modals in a belief context. Being native speakers of German, we focus on examples like (1) with the modal *möglicherweise* ‘possibly’. Example (2) provides a scenario where (1) is judged true.1Examples are glossed following the Leipzig Glossing Rules. Abbreviations used: 3 = third person, aux = auxiliary, imp = imperative, ind = indicative, inf = infinitive, part = particle, prs = present, pst = past, ptcp = participle, refl = reflexive pronoun, sbjv = subjunctive, sg = singular.

(1)

**Table:** 

*Anna glaubt,*	*dass*	*sie*	*möglicherweise*	*einen*	*Kaffeekocher*	*bekommen*
Anna believe.prs.ind.3sg	that	she	possibly	a	coffeemaker	receive.ptcp

**Table:** 

*hätte,*	*wenn*	*das*	*Paket*	*nicht*	*gestohlen worden*	*wäre.*	
aux.pst.sbjv.3sg	if	the	package	not	steal.ptcp aux.ptcp	aux.pst.sbjv.3sg	
‘Anna believes that she would possibly have received a coffeemaker if the package had not been stolen.’

(2)

**Table:** 

scenario: Last week, Anna intended to order two things online: a toaster and a coffeemaker. But ultimately, she ordered only one thing and forgot which one. This week, she was notified that a package had been delivered. When she went to pick it up, she learned it was stolen. She has no idea whether the package contained a toaster or a coffeemaker. It actually contained a toaster.
(1) **true**

Given this scenario, it is plausible to ascribe an epistemic or doxastic flavor to *möglicherweise*.2For an introduction to German modals and the different flavors available to modal adverbs, see Kratzer (1978, 2012 [1981]a). The modal seems to be evaluated relative to Anna’s beliefs about what would have happened if the package had not been stolen, rather than the actual facts determining the most likely scenario: given the facts in scenario (2) where the package actually contains a toaster, a world where Anna receives a coffeemaker would be quite far-fetched, but this seems to be irrelevant to the truth value of (1), which suggests that the counterfactual in (1) is evaluated relative to Anna’s doxastic state.

We will show that such data have unexpected consequences for the analysis of counterfactuals and of belief attributions within possible-worlds semantics (see e.g. Lewis 1973; Kratzer 1978, 2012 [1981]b for the former; Hintikka 1969 for the latter). They reveal a conflict between two widespread assumptions: first, that the quantificational domain of an epistemic modal in a belief context does not vary between the subject’s doxastic alternatives, and second, that counterfactuals quantify over a set of “closest” worlds in which the antecedent holds, where “closeness” is determined by the facts of the evaluation world.

In (1), *möglicherweise* is restricted by a counterfactual and simultaneously occurs in a belief context. The second assumption then predicts that the domain of the modal should vary across the subject’s doxastic alternatives, while the first assumption predicts that it should not. We will argue that to derive plausible truth conditions for (1), the second assumption should be weakened: the orderings between worlds that counterfactuals are sensitive to are not based on similarity to a particular world, but on “closeness” to an information state that may be equally compatible with multiple worlds. In other words, the orderings do not have to satisfy *centering* in the sense of Lewis (1973); they do not necessarily have a unique minimum. This permits us to interpret *möglicherweise* in (1) with respect to an ordering that represents Anna’s belief state as a whole, rather than a particular belief world. In addition to this weakening of the standard semantics of counterfactuals, the puzzle also motivates a revision of Hintikka’s (1969) attitude semantics.

Our conclusion – that worlds are the wrong kind of parameter for the similarity relation underlying counterfactuals – is not completely new. We find related claims in the literature. First, Arregui (2008) uses data involving tense in counterfactuals to argue that worlds are not the right level of granularity, but defends a notion of similarity relativized to situations, not information states. Second, Schulz (2007) argues that counterfactuals sometimes involve a global notion of belief revision, which means they must be interpreted relative to an epistemic state. However, she adopts this type of analysis only for so-called epistemic counterfactuals, whereas we argue it should apply to all counterfactuals to account for their behavior in embedded contexts. Given space limitations, we leave a detailed comparison of our argument with these previous proposals to future work.

## 2 The problem

Epistemic modals in belief contexts usually range over the attitude subject’s doxastic alternatives. However, (1) introduces an additional complication: assuming Kratzer’s (1978, 2012 [1981]a) restrictor approach to conditionals, the domain of *möglicherweise* appears to be restricted by the counterfactual. This raises a puzzle for the traditional view of counterfactuals, on which they quantify over a set of worlds determined by the facts of the evaluation world together with the antecedent proposition. If so, a modal embedded under *believe*, as in (1), may have a different domain in each world *w* among the attitude subject’s doxastic alternatives, depending on the facts of *w*. This variability leads to incorrect truth conditions for sentences like (1).

### 2.1 Standard assumptions about modals in belief contexts

We first consider non-counterfactual epistemic modals embedded under attitudes. Yalcin (2007) observes that conjunctions of an epistemically modalized sentence with the negation of the corresponding non-modalized sentence are contradictory – even in embedded contexts. This observation extends to the embedding of *möglicherweise* under *glauben* ‘believe’, as in (3).3
Yalcin (2007) suggests that his observations concerning the unacceptability of sentences like *Suppose it is raining and it might not be raining* should extend to embedding under *believe*, but does not give examples.

(3)

**Table:** 

#*Anna*	*glaubt,*	*dass*	*sie*	*möglicherweise*	*einen*	*Kaffeekocher*	*bekommen*	*wird*
Anna	believe.prs.ind.3sg	that	she	possibly	a	coffeemaker	receive.inf	will.ind.3sg

**Table:** 

*und*	*dass*	*sie*	*keinen*	*Kaffeekocher*	*bekommen*	*wird.*				
and	that	she	no	coffeemaker	receive.inf	will.ind.3sg			
‘Anna believes that she will possibly receive a coffeemaker and that she will not receive a coffeemaker.’										

Following Hintikka (1969), we take *believe* to quantify universally over the subject *x*’s doxastic alternatives, as determined by an accessibility relation dox(*x*). Without further assumptions about dox(*x*), we get the truth conditions in (4), according to which Anna believes she will not receive a coffeemaker, but also believes that she considers it possible that she will receive a coffeemaker.

(4)

**Table:** 

[[(3)]](*w*_0_) = 1 iff ∀*w*[*w* ∈ dox(**Anna**)(*w*_0_) → ¬∃*y*[**coffeemaker**(*w*)(*y*) ∧ **receive**(*w*)(*y*)(**Anna**)] ∧
∃*w*′[*w*′ ∈ dox(**Anna**)(*w*) ∧ ∃*y*[**coffeemaker**(*w*′)(*y*) ∧ **receive**(*w*′)(*y*)(**Anna**)]]]

Example (3) would correctly come out as contradictory if *möglicherweise* under a doxastic construal had to be interpreted as ranging over exactly those worlds that ‘believe’ quantifies over, or a subset thereof. This can be achieved by blocking the possibility that a subject might be unaware of a belief she holds, or equivalently, requiring dox(*x*) to be transitive:

(5)

**Table:** 

For any worlds *w*, *w*′ such that *w*′ ∈ dox(*x*)(*w*), dox(*x*)(*w*′) ⊆ dox(*x*)(*w*).

If *möglicherweise* receives a doxastic construal, (5) guarantees that for any *w* among Anna’s doxastic alternatives in *w*_0_, *möglicherweise* when evaluated at *w* only ranges over worlds that are themselves among her doxastic alternatives in *w*_0_. The truth conditions in (4) then become contradictory: while the first embedded conjunct states that Anna will not receive a coffeemaker in any of her doxastic alternatives, the second conjunct entails that she will receive a coffeemaker in at least one of them.

In sum, (3) suggests that *möglicherweise* in a belief context usually quantifies over a subset of the belief subject’s doxastic alternatives.4Yalcin’s more general approach to this phenomenon does not rely on a transitive accessibility relation. See Section 3. But this assumption cannot literally extend to counterfactual cases like (1): in scenario (2), the package was stolen in each of Anna’s doxastic alternatives, thus the worlds over which *möglicherweise* quantifies cannot be among these alternatives. So what happens in counterfactuals?

### 2.2 Some previous assumptions about counterfactuals with epistemic modals

We will start with two prima facie plausible assumptions taken from the literature on counterfactuals. First, counterfactuals quantify over a domain of worlds selected in a particular way. Most implementations of this domain-selection mechanism are based either on ordering semantics (Lewis 1973) or on premise semantics (Kratzer 1978). On both approaches, the consequent of a counterfactual is evaluated in those worlds that count as “closest” to the evaluation world among the worlds verifying the antecedent.5To simplify the discussion, we ignore scenarios in which there are no closest worlds; see Lewis (1973, 1981. What counts as “closest” is determined by the facts and generalizations holding in the evaluation world; following Kratzer (1978, 1991, 2012 [1981]a), these can be modeled as a set of propositions that jointly uniquely characterize the world in question.6These propositions probably differ in their status; see Lewis (1979) for discussion of temporal asymmetries among facts and Kaufmann (2005), Schulz (2007), Kaufmann (2013), and others for analyses in which causal generalizations and contingent facts do not have equal weight. Our claim below – that the relevant set of propositions does not have to determine a unique world – is independent of the question whether further subdivisions within this set are needed. However, it is not obvious how to integrate the causal network formalism employed by Kaufmann and Schulz into our analysis – a question we must leave to future work.


The second assumption is perhaps less standard: following the work of Kratzer (1978, 1991, 2012 [1981]a) on conditionals in general, we take counterfactuals with an existential modal like *möglicherweise* to provide an existential quantifier over a set of “closest” worlds verifying the antecedent. The antecedent of a counterfactual then serves to restrict an existential quantifier over worlds. We take the semantic predictions of this analysis for non-embedded counterfactuals to be superior to an account on which the counterfactual involves a covert universal modal that scopally interacts with *möglicherweise*: If *möglicherweise* were to take scope in the consequent of a universally quantified counterfactual, the paraphrase for (6a) would be (6b). However, (6b) requires Anna’s doxastic state in each of the closest worlds in which the package was not stolen to be compatible with her receiving a coffeemaker. This is implausible: if the closest antecedent-worlds included worlds in which she ordered and received a toaster before the utterance time, then in these worlds she would not be uncertain about what she ordered.7Evaluating *möglicherweise* at *w*_0_ will not help as it would make the counterfactual vacuous.

(6)

**Table:** 

a.	*Wenn*	*das*	*Paket*	*nicht*	*gestohlen*	*worden*	*wäre,*	*hätte*	*Anna*
	if	the	package	not	steal.ptcp	aux.ptcp	aux.pst.sbjv.3sg	aux.pst.sbjv.3sg	Anna

**Table:** 

	*möglicherweise*	*einen*	*Kaffeekocher*	*bekommen.*			
	possibly	a	coffeemaker	receive.ptcp			
	‘If the package had not been stolen, Anna might have received a coffeemaker.’									
b.	∀*w*[*w* ∈ closest(*w*_0_)(*λw*′.the package was not stolen in *w*′) → ∃*w*′′[*w*′′ ∈ dox(Anna)(*w*)									
	∧ Anna receives a coffeemaker in *w*′′]]									

Related arguments (which we omit due to space limitations) can be adduced against an analysis on which *möglicherweise* outscopes a counterfactual with a universal modal.

Combined, our assumptions yield the following simplified truth conditions for counterfactuals with *möglicherweise*. We leave the exact nature of the relation closest unspecified, but assume that it appeals to the facts and generalizations holding in *w*_0_.

(7)

**Table:** 

$\left[\left[möglicherweise\left[wenn\text{ p}\right]\text{q}\right]\right]\left(w_{0}\right)=1$ iff ∃w[w∈ closest $\left(w_{0}\right)\left(\left[\left[\text{p}\right]\right]\right)\wedge \left[\left[\text{q}\right]\right]\left(w\right)=1]$

### 2.3 The puzzle

To see why this semantics for counterfactuals clashes with our earlier observations about *möglicherweise* in belief contexts, let us try to interpret (1) by combining (7) with the standard possible-worlds semantics for *glauben* ‘believe’, giving us (8).

(8)

**Table:** 

$\left[\left[\left(1\right)\right]\right]\left(w_{0}\right)=1$ iff ∀*w*[*w* ∈ dox(Anna)(*w*_0_) → ∃*w*′ [*w*′ ∈ closest(*w*)([[p]]) ∧ [[q]](*w*′) = 1]]
where [[p]]=(λw.the package was not stolen in w) and [[q]]=(λw.Anna received a coffeemaker in w)

Here, a counterfactual with the semantics in (7) is evaluated separately in each of Anna’s doxastic alternatives: (8) says that each *w* among these alternatives is such that in one of the closest worlds to *w* in which the package was not stolen, Anna received a coffeemaker. But these truth conditions are empirically inadequate: in scenario (2), Anna has no idea whether the stolen package contained a toaster or a coffeemaker. So there is a doxastic alternative *w*_*t*_ in which it contained a toaster. The truth conditions in (8) then entail that in at least one of the closest worlds to *w*_*t*_ in which the package was not stolen, Anna would nonetheless have received a coffeemaker. But if the closest relation is grounded in the facts of a particular world, how would such a coffeemaker world end up in the set of closest worlds for *w*_*t*_?

While there might be worlds in which Anna ordered a toaster, but it was erroneously replaced with a coffeemaker before the package got stolen, such worlds will be very far-fetched from the perspective of *w*_*t*_ and should thus not be in closest(*w*_*t*_)([[p]]). This is confirmed by the observation that (1) is false in a scenario where Anna is certain she only ordered a toaster. In fact, the need to exclude such exceptional worlds is exactly what motivates use of the closest relation, as opposed to a strict-conditional analysis of counterfactuals, to begin with. Thus, we cannot attribute the acceptability of (1) in scenario (2) to the availability of such worlds.

Rather, the root of the problem seems to be that the closest relation, when evaluated in a world *w* among Anna’s doxastic alternatives, is based on a full set of propositions that uniquely characterize *w*, regardless of whether Anna actually believes these propositions. In Section 3, we will revise this assumption. But first, we address another potential solution: could we not assume, contra Hintikka (1969), that the quantificational force of attitude verbs is not universal8
Hawthorne et al. (2016) provide the most explicit proposal of this type that we are aware of (but see also Lassiter 2017 for a similar intuition): they suggest that a sentence *a believes p* is true iff *a*’s (subjective) confidence in *p* is larger than some contextual threshold. See Koev (2019) for a critical assessment. and weaken the analysis of belief so that it does not require us to consider any worlds in which the package contains a toaster? We cannot argue against all potential versions of weak belief here, but there is one reason why this is probably not the right explanation for the acceptability of (1) in scenario (2): when *möglicherweise* is replaced with the stronger epistemic modal *bestimmt* ‘definitely’, the resulting sentence (9) is no longer true in the scenario. If we could ignore the “toaster worlds” among the doxastic alternatives, so that the package contains a coffeemaker in all the doxastic alternatives under consideration, (9) and (1) should be equally acceptable.

(9)

**Table:** 

*Anna*	*glaubt,*	*dass*	*sie*	*bestimmt*	*einen*	*Kaffeekocher*	*bekommen*	*hätte,*
Anna	believe.prs.ind.3sg	that	she	definitely	a	coffeemaker	receive.ptcp	aux.pst.sbjv.3sg

**Table:** 

*wenn*	*das Paket*	*nicht*	*gestohlen*	*worden*	*wäre.*			
if	the package	not	steal.ptcp	aux.ptcp	aux.pst.sbjv.3sg			
‘Anna believes that she would definitely have received a coffeemaker if the package had not been stolen.’							
**false** in (2)									

We therefore turn to our preferred option – revising the role of the closest relation.

## 3 Proposal

The basic idea is that, for counterfactuals embedded under *believe*, the relevant set of closest worlds making the antecedent true is not determined on the basis of the facts of a particular world. Rather, it is based on the attitude subject’s doxastic state as a whole. This doxastic state provides an ordering among worlds which must satisfy *weak centering* in the sense of Lewis (1973), but not centering; that is, it must have minimal elements, but these do not have to be unique.9Strictly speaking, weak centering in Lewis’s sense also requires the evaluation world that determines the choice of ordering to be one of the minimal elements in the ordering. Since we take the ordering to be determined by the matrix evaluation world *w*_0_, this typically will not hold, as *w*_0_ will not generally be compatible with subjects’ belief states in *w*_0_. However, there is a sense in which our belief states satisfy the spirit (although not the letter) of Lewis’s definition: below, we will assume that *believe* quantifies over complex indices $〈w,\underset{‾}{≺}〉$ such that the set of $\underset{‾}{≺}$ -minimal worlds always includes *w*.


### 3.1 The role of information states

To spell this out, we first return to (8) and consider what the closest relation would have to look like to correctly predict that (1) is true in scenario (2). Let *w*_*t*_ be one of Anna’s doxastic alternatives in which her package contained a toaster, and *w*_*c*_ an alternative where it contained a coffeemaker. If the counterfactual is evaluated separately in each of *w*_*t*_ and *w*_*c*_, we get the right truth conditions only if closest(*w*_*t*_)([[p]]) and closest(*w*_*c*_)([[p]]) each contain worlds in which Anna actually received a coffeemaker. We are then ultimately giving up the intuition that the similarity relation accessed by counterfactual uses of *möglicherweise* is rooted in the facts and generalizations holding in a particular world. If all the facts of *w*_*t*_ are taken into account – including the fact that the package contained a toaster – a world in which Anna received a coffeemaker just cannot be maximally similar to *w*_*t*_.

However, there is a natural way of characterizing the truth conditions of (1) that preserves the idea that counterfactuals involve similarity relations. Consider the notion of a similarity-based ordering ${\underset{‾}{≺}}_{w}$ from Lewis (1973), where $w^{\prime }{\underset{‾}{≺}}_{w}w^{\prime \prime }$ means that *w*′ is at least as similar to *w* as *w*′′ is. If these orderings are based on the facts and generalizations holding in a world *w*, they must be *centered*: there must be a unique minimal element, *w* itself.

Let us now think of Anna’s belief state in *w*_0_ as a consistent set *S* of propositions whose intersection is the set dox(Anna)(*w*_0_).10As discussed by Kratzer (1978, 2012 [1981]b) for non-embedded conditionals, there will be many such sets that pick out the same doxastic alternatives. The problem of choosing between multiple premise sets arises in all versions of premise semantics. Worlds could then be partially ordered by a relation ${\underset{‾}{≺}}_{\text{Anna},w_{0}}$ , where $w{\underset{‾}{≺}}_{\text{Anna},w_{0}}w^{\prime }$ iff *w* comes at least as close as *w*′ to satisfying all the propositions in *S*. The exact definition of this ordering is irrelevant for our purposes (but see in particular Lewis 1981; Kaufmann 2005, 2013; Schulz 2007). But importantly, in Lewis’s (1973) terminology, it will be weakly centered, but not centered, that is, it may have multiple minimal elements: a world *w* will be a minimal element if and only if *w* satisfies all the propositions in *S*. So Anna’s doxastic alternatives are the minimal elements of *S*.

We submit that, when a counterfactual with a modal like *möglicherweise* is evaluated in the context of Anna’s beliefs in *w*_0_, the modal’s quantificational domain does not vary across doxastic alternatives, but is determined by the ordering ${\underset{‾}{≺}}_{\text{Anna},w_{0}}$ which encodes Anna’s doxastic state as a whole.11That belief states can be related to similarity orderings of the kind needed to interpret counterfactuals is not a new idea in the context of belief revision. A reviewer mentions Harper (1976), which we did not have access to at the time of writing; another reviewer points out a connection with Grove (1988), who uses Lewis’s notion of a weakly centered similarity ordering to model belief revision, and, also in the context of belief revision, Gärdenfors (1988). Given these connections, let us clarify that we do not analyze counterfactuals in general as involving belief revision or as sensitive to belief states regardless of the context they occur in. While counterfactuals embedded under overt belief predicates are sensitive to belief states, we do not assume this for counterfactuals embedded under other attitudes. The domain will consist of the minimal worlds in ${\underset{‾}{≺}}_{\text{Anna},w_{0}}$ that satisfy the antecedent. The resulting truth conditions for (1) are paraphrased in (10a), where the closest relation is defined as in (10b). Note that, since the modal’s domain remains constant across doxastic alternatives, the universal quantifier contributed by *believe* is vacuous.

(10)

**Table:** 

a.	$\left[\left[\left(1\right)\right]\right]\left(w_{0}\right)=1$ iff ∀*w*[*w* ∈ dox(Anna)(*w*_0_) → ∃*w*′[*w*′ ∈ closest $\left({\underset{‾}{≺}}_{\text{Anna},w_{0}}\right)\left(\left[\left[\text{p}\right]\right]\right)\wedge \left[\left[\text{q}\right]\right]\left(w^{\prime }\right)=1]]$
	where [[p]]=(λw.the package was not stolen in w) and [[q]]=(λw.Anna received a coffeemaker in w)
b.	For a partial ordering $\underset{‾}{≺}$ among worlds and a proposition *p*,
	closest $\left(\underset{‾}{≺}\right)\left(p\right)=\left\{w\in \text{dom}\left(\underset{‾}{≺}\right)|p\left(w\right)=1\wedge \neg \exists w^{\prime }\left[w^{\prime }≺w\wedge p\left(w^{\prime }\right)=1\right]\right\}$

How does this solve our original puzzle? Consider a world *w*_0_ described by scenario (2). Among Anna’s doxastic alternatives, there are “toaster worlds” *w*_*t*_ and “coffeemaker worlds” *w*_*c*_. Further, there is a world $w_{t^{\prime }}$ that differs from *w*_*t*_ only in that the package was not stolen; in particular, the package in $w_{t^{\prime }}$ contains a toaster. Similarly, there is a “coffeemaker world” $w_{c^{\prime }}$ minimally different from *w*_*c*_ in which the package was not stolen. The new closest relation in (10b) has us consider the lowest-ranked worlds in ${\underset{‾}{≺}}_{\text{Anna},w_{0}}$ where the package was not stolen. Since Anna has no idea whether there was a toaster or a coffeemaker in the package, we can assume that neither of $w_{c^{\prime }}$ and $w_{t^{\prime }}$ ranks lower than the other. This corresponds to the intuition that $w_{c^{\prime }}$ and $w_{t^{\prime }}$ come equally close to satisfying all of Anna’s beliefs. So (1) is correctly predicted true in scenario (2).

In sum, we propose to account for the weak semantics of existential counterfactuals in belief contexts by weakening the centering assumption adopted in Lewis’s (1973) ordering semantics, or equivalently adopting a weaker version of premise semantics where the premises do not jointly characterize a unique world: The orderings we use to interpret counterfactuals are based on an information state that may leave certain issues undetermined. Two worlds that differ only on one of these undetermined issues may then be incomparable, whereas the standard semantics of counterfactuals would require us to order one world below the other, depending on how the issue is resolved in the evaluation world.

### 3.2 An implementation based on Yalcin’s (2007) domain semantics

Given our proposal that the ordering relation exploited by counterfactuals is not always grounded in the facts of a particular world, the universal modal quantification introduced by ‘believe’ in (10a) is vacuous. This aspect of our paraphrase is crucial to account for the coffeemaker puzzle, but it raises a compositionality problem: if the counterfactual is evaluated relative to an ordering relation that depends on the evaluation world *w*_0_, and ‘believe’ shifts the world parameter to worlds other than *w*_0_ in the usual way, how does the counterfactual get access to this ordering?12A reviewer suggests this problem could be solved by appealing to the introspection properties of belief: Natural language semantics seems to treat subjects as having full knowledge of their own belief states (but see Hintikka 1962). We could therefore assume that in each of Anna’s doxastic alternatives relative to *w*_0_, her belief state must be the same as in *w*_0_. Extending this idea to counterfactual beliefs would mean that ${\underset{‾}{≺}}_{\text{Anna},w_{0}}={\underset{‾}{≺}}_{\text{Anna},w}$ must hold for each doxastic alternative *w*. Each of the doxastic alternatives would thus make Anna’s belief state in *w*_0_ accessible. However, there is an empirical reason to prefer our grammatical approach: The domain of epistemic modals is not always determined by a belief state; under other attitudes, the relevant information state is determined by the embedding attitude (Yalcin 2007). For instance, if an epistemic modal is embedded under verbs meaning ‘suppose’ or ‘imagine’, it seems to quantify over worlds compatible with the imagined situation, not over worlds compatible with the subject’s beliefs in the imagined situation. This is illustrated by the minimal pair in (i), adapted from Yalcin (2007): (ia) demonstrates that one can consistently suppose that some proposition *p* is true and that one considers ¬p epistemically possible. Yet, this reading cannot be expressed using *might* (ib). The unacceptability of (ib) follows immediately if the embedding attitude shifts the domain of *might* to a set of worlds compatible with the subject’s supposition, as sketched for *believe* in the main text.

(i)

**Table:** 

a.	*Suppose Max is lying to you and you do not believe he is lying to you*.
b.	#*Suppose Max is lying to you and he might not be lying to you*.

As (ii) shows, German *möglicherweise* behaves like *might* in (i) when embedded under *sich vorstellen* ‘imagine, suppose’.

(ii)

**Table:** 

#*Stell*	*dir*	*vor*,	*der*	*Max*	*würde*	*dich*	*belügen*	*und*	*er*	*würde*	*dich*	*möglicherweise*	*nicht*
imagine.imp	refl	part	the	Max	aux.sbjv.prs.3sg	you	lie.to	and	he	aux.sbjv.prs.3sg	you	possibly	not
*belügen*.													
lie.to													
‘Suppose/imagine Max is lying to you and he is possibly not lying to you.’

Generally speaking, such data suggest that we need to model a dependency between *möglicherweise* and the embedding attitude, regardless of the introspective properties of that attitude. (Iterating *suppose* or German *vorstellen* does not give rise to the same introspection behavior as iterating *believe/glauben* either.) That being said, embedded *möglicherweise* is restricted in ways that are not well understood.


We submit that some expressions, including counterfactuals, are sensitive to an information state that acts as a separate parameter of the semantic interpretation function, independently of the world parameter. The truth value of sentences containing such expressions will in general not be determined by the evaluation world. To implement this idea without committing to premise semantics, we model information states as partial orderings among worlds. For instance, a belief state will be a partial ordering with the subject’s doxastic alternatives as minimal elements; the interpretation of counterfactuals relative to this state will depend on the arrangement of the non-minimal worlds in the ordering. This provides us with an analysis of *believe* that extends to cases like (1): *believe* manipulates both the world parameter and the information-state parameter, shifting the latter to the ordering representing the subject’s belief state.

This idea is a slight adaptation of Yalcin’s (2007) proposal about epistemic modals in attitude contexts (see Section 2.1). Yalcin (2007) argues that the indices passed to the interpretation function are pairs consisting of a world and what he calls an *information parameter*, which he represents as an unordered set of worlds. This has two consequences: First, the denotations of expressions can be sensitive to different components of complex indices; for example, epistemic modals only consider the information parameter. Second, the semantics of expressions can shift or quantify over the components of complex indices individually. Yalcin proposes that attitude verbs shift both components: they quantify over different potential values of the world parameter, but also shift the information parameter (in the case of *believe*, to the subject’s epistemic state).

We adopt the general structure of this proposal, but generalize Yalcin’s notion of an information parameter: Since this parameter will provide the quantificational domain for counterfactuals, unordered sets of worlds will be insufficient. Instead, we define indices – the elements of *D*_*s*_ – as ordered pairs $i=〈w_{i},{\underset{‾}{≺}}_{i}〉$ , with *w*_*i*_ a world and ${\underset{‾}{≺}}_{i}$ a partial ordering among worlds that represents an information state.

Given this richer notion of indices, we will now develop an analysis of (1). For expressions like (11a), which contain no modals, counterfactuals or similar expressions, our semantics does not differ significantly from the traditional view. The intension of (11a) is a function from complex indices to truth values, but this function is insensitive to the information-state component of the index.

(11)

**Table:** 

a.	*The package wasn’t stolen.*
b.	[[(11a)]] = $\lambda i.\text{the package was not stolen in }w_{i}$ .

Next, we consider counterfactuals with existential modals. Without going into their internal composition, we assume following Kratzer (1978, 1991, 2012 [1981]b) that they quantify existentially over the minimal worlds in the relevant ordering which make the antecedent true. However, this ordering is now determined by the information state ${\underset{‾}{≺}}_{i}$ of the evaluation index *i*. (In the unembedded case, this information state could arguably be determined by the common ground [see Yalcin 2007 for discussion], so that the ordering’s minimal elements would constitute the context set.) Example (12) shows that the counterfactual selects the minimal elements relative to ${\underset{‾}{≺}}_{i}$ among the worlds making the antecedent true. The consequent is evaluated at indices consisting of one of these worlds and ${\underset{‾}{≺}}_{i}$ itself. Hence, the counterfactual quantifies over a domain determined by the information-state component of its index and completely ignores the world component.

(12)

**Table:** 

a.	*possibly* [*if* [_*p*_ *the package had not been stolen*]] [_*q*_ *Anna would have received a coffeemaker*]
b.	$\begin{array}{l} \left[\left[\left(12\text{a}\right)\right]\right]=\\ \lambda i.\exists w\left[\left[w\in \text{dom}\left({\underset{‾}{≺}}_{i}\right)\wedge \left[\left[\text{p}\right]\right]\left(\left\langle w,{\underset{‾}{≺}}_{i}\right\rangle \right)=1\wedge \neg \exists w^{\prime }\left[w^{\prime }{≺}_{i}w\wedge \left[\left[\text{p}\right]\right]\left(\left\langle w^{\prime },{\underset{‾}{≺}}_{i}\right\rangle \right)=1\right]\right]\wedge \left[\left[\text{q}\right]\right]\left(\left\langle w,{\underset{‾}{≺}}_{i}\right\rangle \right)=1\right] \end{array}$

The final prerequisite is a revised semantics for *believe* (13), which has two jobs: to perform a shift to an information state ${\underset{‾}{≺}}_{x,w_{i}}$ that encodes the subject’s beliefs in the evaluation world, and to quantify over indices consisting of this new information state and some doxastic alternative of the subject’s. Notice that (13) appeals to the minimal elements of ${\underset{‾}{≺}}_{x,w_{i}}$ (in a global sense, not relative to some antecedent proposition), which are the subject’s doxastic alternatives.

(13)

**Table:** 

$\begin{array}{l} \left[\left[believe\right]\right]=\\ \lambda i.\lambda p_{〈s,t〉}.\lambda x_{e}.\forall w\left[\left[w\in \text{dom}\left({\underset{‾}{≺}}_{x,w_{i}}\right)\wedge \neg \exists w^{\prime }\left[w^{\prime }{≺}_{x,w_{i}}w\right]\right]\to p\left(〈w,{\underset{‾}{≺}}_{x,w_{i}}〉\right)\right] \end{array}$

For complements that do not depend on the information-state component of an index, (13) preserves Hintikka’s (1969) intuition that *believe* quantifies universally over doxastic alternatives: in (14), the proposition expressed by the embedded clause is evaluated at each index consisting of Anna’s belief state and a doxastic alternative.

(14)

**Table:** 

a.	*Anna believes that the package wasn’t stolen.*
b.	$\left[[\left(14\text{a}\right)]]=\lambda i.\forall w[[w\in \text{dom}\left({\underset{‾}{≺}}_{\text{Anna},w_{i}}\right)\wedge \neg \exists w^{\prime }[w^{\prime }{≺}_{\text{Anna},w_{i}}w]\right]$
	→the package was not stolen in w]

Embedding the counterfactual in (12a) under *believe* finally yields our motivating example (1). As (15) shows, *believe* quantifies universally over indices with the information-state component shifted to ${\underset{‾}{≺}}_{\text{Anna},w_{i}}$ and the world component shifted to one of Anna’s doxastic alternatives. The counterfactual denotation (12b) is evaluated at each of these indices. Importantly, the truth conditions of this counterfactual do not depend on the world component of its evaluation index; it simply quantifies over the minimal worlds in the respective information state at which the package was not stolen. Since all the indices *believe* quantifies over have the same information-state component, quantification over the world variable *w* in (15) is vacuous and the truth conditions reduce to a single instance of existential quantification over worlds. Thus, (15) expresses the desired truth conditions: in some of the minimal worlds relative to ${\underset{‾}{≺}}_{\text{Anna},w_{i}}$ in which the package was not stolen, Anna receives a coffeemaker.

(15)

**Table:** 

$\left[\left[\left(1\right)\right]\right]=\lambda i.\forall w\left[\left[w\in \text{dom}\left({\underset{‾}{≺}}_{\text{Anna},w_{i}}\right)\wedge \neg \exists w^{\prime }\left[w^{\prime }{≺}_{\text{Anna},w_{i}}w\right]\right]\to \left[\left[\left(12\text{a}\right)\right]\right]\left(〈w,{\underset{‾}{≺}}_{\text{Anna},w_{i}}〉\right)\right]$
= $\lambda i.\forall w[[w\in \text{dom}\left({\underset{‾}{≺}}_{\text{Anna},w_{i}}\right)\wedge \neg \exists w^{\prime }[w^{\prime }{≺}_{\text{Anna},w_{i}}w]]$
$\to \exists w^{\prime }[w^{\prime }\in \text{dom}\left({\underset{‾}{≺}}_{\text{Anna},w_{i}}\right)\wedge \text{the package was not stolen in }w^{\prime }\wedge$
$\neg \exists w^{\prime \prime }[w^{\prime \prime }{≺}_{\text{Anna},w_{i}}w^{\prime }\wedge \text{the package was not stolen in }w^{\prime \prime }]\wedge \text{Anna received a coffeemaker in }w^{\prime }]]$
= $\lambda i.\exists w^{\prime }[w^{\prime }\in \text{dom}\left({\underset{‾}{≺}}_{\text{Anna},w_{i}}\right)\wedge \text{the package was not stolen in }w^{\prime }\wedge$
$\neg \exists w^{\prime \prime }[w^{\prime \prime }{≺}_{\text{Anna},w_{i}}w^{\prime }\wedge \text{the package was not stolen in }w^{\prime \prime }]\wedge \text{Anna received a coffeemaker in }w^{\prime }]$

Let’s sum up the idea behind this analysis. We argued in Section 2 that counterfactuals in belief contexts should not be sensitive to all the facts characterizing a particular doxastic alternative. They should only consider propositions actually believed by the subject. We suggested that, while counterfactuals are sensitive to an ordering among worlds, this ordering may be determined by an information state that leaves certain issues unresolved and therefore does not have to be centered, only weakly centered (Lewis 1973). Our implementation dissociates the truth conditions of counterfactuals from the evaluation world, following Yalcin’s (2007) analogous claim about epistemic modals. Instead, counterfactuals (and epistemic modals) depend on a separate parameter passed to the interpretation function. We took the possible values of this parameter to be orderings among worlds, although one could also use Kratzer’s (2012 [1981]b) *conversational backgrounds*. Essentially, our claim is that embedded uses of counterfactuals reveal that counterfactuals and epistemic modal constructions are more similar than they might seem: neither requires the information state used to select the domain for the modal to characterize a unique world.
